# Editorial: Coenzyme Q Redox State and Cellular Homeostasis

**DOI:** 10.3389/fphys.2018.00912

**Published:** 2018-07-30

**Authors:** Alberto Sanz, Placido Navas

**Affiliations:** ^1^Institute for Cell and Molecular Biosciences, Newcastle University Institute for Ageing, Newcastle University, Newcastle upon Tyne, United Kingdom; ^2^Centro Andaluz de Biología del Desarrollo, Universidad Pablo de Olavide-CSIC, and CIBERER, ISCIII, Seville, Spain

**Keywords:** Coenzyme Q, ubiquinol, ubiquinone, mitochondria, cellular homeostasis

Coenzyme Q (CoQ) is a ubiquitous component of eukaryotic lipid membranes, where it participates in electron transfer and acts as a powerful antioxidant. CoQ has a benzoquinone ring and a polyisoprenoid tail that contains between 6 and 10 units depending on the species e.g., six in *Saccharomyces cerevisiae* and 10 in *Homo sapiens*. CoQ has the capacity to accept and donate electrons and in cells is found in three different redox states: (i) oxidized (ubiquinone), semioxidized (semiubiquinone) and reduced (ubiquinol). Although CoQ is usually synthetized within the cell, it is possible to supplement this through diet. This characteristic has made CoQ one of the bestselling dietary supplements. Mutations in genes that encode for the multiprotein CoQ synthesis complex result in severe inherited diseases. Furthermore, mitochondrial diseases resulting from mutations in nuclear or mitochondrial encoded genes are often characterized by a secondary CoQ deficiency. A reduction in CoQ levels has also been observed during aging and in many other non-hereditary diseases (Yubero et al., 2016). In this special issue, we have brought together a selection of the leading scientists in the field to discuss the latest developments in understanding how CoQ contributes to the maintenance of cellular homeostasis.

CoQ has been put forward as an anti-aging supplement as well as a potential treatment for several human diseases. In this special issue, Hernandez-Camacho and collaborators (Hernandez-Camacho et al.) have reviewed the latest literature reporting the effects of CoQ supplementation on human health. The authors discuss studies reporting positive effects or lack thereof on aging and age-related pathologies. CoQ supplementation has been reported to alleviate disorders caused by mutations in the endogenous CoQ synthesis machinery (Hernandez-Camacho et al.). However, positive effects of CoQ supplementation in other disorders have also been reported (Hernandez-Camacho et al.). For example, modest improvements have been described in cardiovascular disease (the number one cause of death in western society) and neurodegenerative disorder (e.g., Parkinson's disease). In addition, treatment with CoQ has been shown to have anti-inflammatory effects and a number of published reports suggest that combining CoQ with statins could prevent the side effects resulting from statin treatment.

Although, CoQ supplementation is the standard treatment for patients suffering from CoQ deficiencies, not all patients respond to this intervention. This is due in part to the poor bioavailability of CoQ. Fabien Pierrel (Pierrel) discusses the possibility of using analogs of a CoQ precursor, 4-hydroxybenzoic acid, as a therapeutic alternative to restore electron flow in the inner mitochondrial membrane. To facilitate this, a better knowledge of the mechanisms responsible for CoQ biosynthesis (CoQ-synthome) and how the complex is regulated is necessary.

Free radicals have long been thought to be responsible for aging and age-related diseases (Sanz et al., 2006). However, recent reports indicate they play a more complicated role than previously anticipated, with both positive and negative effects on longevity (Sanz, 2016). The redox state of CoQ plays an essential role in determining where and how electrons leak from the electron transport chain producing Reactive Oxygen Species (ROS) (Murphy, 2009). Scialo and collaborators (Scialo et al.) discuss how the redox state of CoQ impacts on whether ROS are produced in the forward or reverse direction by respiratory complex I and how ROS produced through reverse electron transport control important physiological functions including regulation of lifespan.

Mounting the appropriate response to various stresses is an essential component of cellular homeostasis. NAD(P)H:quinone acceptor oxidoreductase1 (NQO1) plays a central role in processes of stress adaptation including oxidative stress (Forthoffer et al., 2002). Ross and Siegel review how NQO1 regulates the stress adaptation response as a central component of the Plasma Membrane Redox System that is responsible for keeping lipid peroxidation in line, reducing oxidized antioxidants such as CoQ and vitamin E (Villalba et al., 1995).

CoQ is the electron carrier which connects enzymes that introduce electrons into the mitochondrial electrons transport chain to those that remove them. The former includes many enzymes that participate in important metabolic pathways such as iron sulfur cluster synthesis, nucleotide synthesis and sulfide metabolism (Lill et al., 2012; Quinzii et al.). Quinzii and col (Quinzii et al.) explain the effects of CoQ deficiency on sulfide metabolism. One of the essential regulators of sulfide metabolism is sulfide:quinone oxidoreductase (SQOR) that oxidizes hydrogen sulfide transferring electrons to mitochondrial ubiquinone. Consequently, deficits in CoQ biosynthesis interrupt hydrogen sulfide oxidation and the production of glutathione. Furthermore accumulation of hydrogen sulfide can cause inhibition of respiratory complex IV further disrupting electron transport.

Finally, we have an original article where He and collaborators (He et al.) use the power of yeast genetics to study the role of COQ9 in the assembly and stability of the CoQ-synthome i.e. the multi-subunit complex that synthetizes CoQ in the mitochondrion. The authors perform a careful study where they interrogate the ability of human COQ9 to rescue mutations in its yeast homolog. They authors demonstrate that COQ9 restores CoQ biosynthesis under specific circumstances by increasing the stability of other components of the CoQ-synthome in *coq9* mutants, but also show that certain properties of *coq9* (e.g., editing CoQ-intermediates to remove amino groups) are not conserved. The approach used by He and collaborators can be extended to other genes involved in CoQ metabolism and mutated in human disease whose function has not been fully characterized.

## Author contributions

AS and PN were in charge of the review process of the papers published in the Research Topic of Frontiers entitled The Redox State of Coenzyme Q and Cellular Homeostasis that are summarized in this editorial.

### Conflict of interest statement

The authors declare that the research was conducted in the absence of any commercial or financial relationships that could be construed as a potential conflict of interest.
